# My A–T pack: a qualitative study of the utility, acceptability, design, and content of a family-designed and owned information pack relevant to the lives of children and young people living with ataxia telangiectasia

**DOI:** 10.1186/s13023-025-03919-6

**Published:** 2025-08-04

**Authors:** Munira Khan, Elizabeth Cassidy, Tracey Parkin, Amanda Wallace, Bernie Carter, William Whitehouse, James Munro, Joanne Paton, A.-Team Collaborative, Lisa Bunn

**Affiliations:** 1 Faculty of Health, University of Plymouth, Plymouth, UK; 2 Freelance Academic, London, UK; 3 Edge Hill University, Ormskirk, UK; 4 National Paediatric Ataxia Telangiectasia Clinic, Nottingham Children’s Hospital, Nottingham, UK; 5 Mistermunro, Liverpool, UK; 6 Cicely Saunders Institute of Palliative Care, Policy & Rehabilitation, King’s College London, London, UK

**Keywords:** Ataxia telangiectasia, Cerebellar ataxia, Multidisciplinary management, Multidisciplinary care, Patient held health records, Patient held notes, Focus groups

## Abstract

**Background:**

Ataxia telangiectasia (A–T) is a rare genetic and progressive condition, primarily affecting the neurological, immunological, and pulmonary systems. In the absence of a cure, people living with A–T require co-ordinated multidisciplinary care to manage their complex needs. This often leads to families working with a range of different professionals and feeling burdened by the amount of information and coordination of care that they manage.

With the aim to inform the co-production of a family-owned healthcare pack to promote person-centered care and self-management, this study explored the views of children with A–T and parents of children and young people with A–T about the utility, acceptability, design, and content of this pack.

**Results:**

A total of two children and eight parents participated in one pilot interview and three focus groups. Using the Framework Method of analysis, three themes were generated offering an insight to the range of participants’ views. The first theme, ‘*accessing, managing, organising, and sharing information with others*’, broadly highlighted the need for a pack as a valuable resource in the absence of coordinated care and a centralised system of record keeping and information sharing. The second theme, ‘*pack content*’, suggested that the pack may serve the dual purpose of storing and retrieving information and helping to communicate and work with other professionals. The third theme, ‘*design features*’, investigated the design of the pack and the differences in the views of children who wanted the pack to look like a magazine style booklet, and the parents who preferred an electronic pack or an app.

**Conclusion:**

This study is an important contribution to the current understanding of the experiences of care and management of A–T from the point of view of children with A–T and their parents. Families with a child with A–T struggle with communication and information sharing across and between different professionals. My A–T Pack is a step towards providing families a viable resource for effective record keeping, symptoms management, and information sharing with relevant professionals involved in the care and management of their child's condition.

**Supplementary Information:**

The online version contains supplementary material available at 10.1186/s13023-025-03919-6.

## Background

Ataxia telangiectasia (A–T) is a rare genetic and progressive condition, usually presenting in early childhood and primarily affecting the neurological, immunological, and pulmonary systems [1]. A–T generally presents at 12–18 months with an unsteadiness of gait due to cerebellar ataxia, and ataxia gradually worsens with children being unable to walk by the age of 10 years [2]. Other problems with oculomotor, extrapyramidal and peripheral nervous system occur in later childhood and adolescence [3]. Lung disease is common and frequently worsens with increasing age and progressive neurological decline [4]. Diseases of the respiratory system cause significant morbidity and are a frequent factor or cause of death [5]. Difficulties with feeding, swallowing and nutrition are common [6], and people with A–T are also at high risk of malignancy [4]. World-wide prevalence estimates vary between 1 in 40,000 and 1 in 100,000 live births [1] and life expectancy is generally limited to 20–30 years of age in people with classical A–T (the severest form of A–T) [2].

In the absence of a cure, people living with A–T require coordinated multi-disciplinary care to optimally manage their complex needs [3]. Symptomatic management and rehabilitation can greatly improve the quality of life of people living with this condition and prevent complications that could increase morbidity and mortality [7]. Despite the potential for allied-health targeted management, parents participating in our patient and public involvement and engagement (PPIE) meeting reported being uncertain about what living with A–T means to family life and the life of their child. They were unclear about how best to deal with A–T, how to find help and how and when to access support. In response to these concerns, the A–Team Collaborative (including family members of children and young people with A–T, therapists, clinicians, and researchers) was convened to undertake a multi-stage project [8] targeted at exploring and promoting therapy management of A–T.

### Patient-held health records

Patient-accessible medical records provide modest improvements in doctor-patient communication, adherence to treatment regimens, patient empowerment, and patient education [9]. Recent trends in information technology have made it easier for patients to access and review their records. Studies have shown that electronic health records are broadly welcomed by patients and healthcare practitioners [10–12]. Some National Health Service (NHS) organisations in the UK are also promoting the use of patient portals (electronic patient record systems) to enable easy access for clinical staff and to encourage patients to take an active role in their healthcare through a programme called ‘MY CARE’ [13].

Patient-held medical records are a more recent innovation in supporting patient-centered healthcare. In this format, patients are given a copy of their records to keep, and to take to health appointments, to help manage healthcare tasks and communication [14]. Patient-held health records (PHRs) exist in a variety of formats such as booklets, logbooks, copies of clinic letters and summaries, and electronic health records [15–17]. PHRs are believed to promote patient-centered care and benefit patients by providing information that can easily be shared with others, promoting a more active role in care and the possibility of contributing to decision making [18–20]. Adopting a patient-centered approach, they promote patient self-management and greater involvement in their own care [20, 21] by making medical records and other relevant information accessible [18]. The benefits of PHRs to children and young people (CYP) should not be underestimated. With growing complexity of needs among CYP, their participation in decision making process in healthcare is essential and therefore should be advocated [22, 23]. The use of PHRs is seen as a potential strategy for improving CYP’s health advocacy skills [19] and involvement in their own care [21].

However, the current versions of the PHRs being used by CYP only partially improve their control over decision making and management of their condition. Their scope in promoting health advocacy skills for CYP is limited by their ownership and use mainly being controlled by the healthcare providers and/or the parents on behalf of their child(ren) [24]. In a realist review of PHRs, Diffin et al. [19] concluded that ‘further research is needed to evaluate the use of PHRs by CYP themselves, in particular those with life‐limiting or life‐threatening conditions’. In addition, CYP should be involved in the design and evaluation of the PHRs to express what matters to them and to have their opinions valued. So far, no such studies have been conducted that involve CYP in co-production of PHRs that are meant for their use. This study has attempted to address this gap by co-producing a version of PHRs for CYP with A–T, which goes beyond simple medical record keeping of the diagnosis, medication, and appointments.

The aim of this study was to inform the co-production of an A–T specific family-owned healthcare pack by gathering the views of children with A–T and parents of CYP with A–T about the utility, acceptability, design, and potential content of this pack.

## Methods

### Research approach

A qualitative research approach was used to gather the experience, ideas and needs of children with A–T and their parents living in the UK, in the development of a family-owned pack of health records. As part of our Patient and Public Involvement and Engagement (PPIE) for the study, we engaged with parents of children with A–T who stated a preference for the use of focus groups over individual interviews as *“working as a team is better than [a] one-to-one approach…meeting in a group would help people share ideas as a group”* (Parent, PPIE). This view aligned with the known strengths of focus groups which would allow both exploration of convergent and divergent views that arise from group interaction [25] and support the collective construction of the pack.

This study has used the ethos of co-production by working in partnership with children with A–T and parents of CYP with A–T, clinicians, and allied health professionals to drive research forward together. Co-production has helped researchers to understand the complex needs and experiences of children and parents, while at the same time both promoting increased confidence and capacity building [26] by offering greater control over the research process, along with opportunities to learn from and reflect on their experiences [27].

### Participants and recruitment

Participants were recruited via social media posts and direct emails sent by the A–T Society charity to families who had expressed an interest in being contacted for research purposes. Purposive sampling was used to recruit individuals who responded to the study advertisement and met the following inclusion criteria: (a) Parents or carers were eligible if they had at least one child aged 5–16 years diagnosed with A–T; (b) Children were eligible if they were aged 8–16 years and diagnosed with A–T.

### Data collection

One pilot interview and three focus group sessions were conducted online to collect data for the co-production of the pack. The pilot session was originally planned as a focus group with three parents; however, due to only one parent attending, it was conducted as an individual interview instead. The remaining sessions were conducted as planned, in the form of focus groups. While this shift in the data collection method deviated from the planned approach for the pilot, the session still served an important purpose. It allowed for testing of the topic guide and refining the phrasing and delivery of the questions, ensuring the guide’s suitability for subsequent sessions.

No changes to the topic guide were identified as necessary from the pilot session. Therefore, the data derived from this interview were deemed relevant and included as part of the final dataset.

The topic guide for the focus group concentrated on need, content, utility, and design of the pack. The pilot interview and focus groups were conducted online via Zoom, each lasting between 30 to 50 min. All the data collection sessions were audio and video recorded following consent of the attendees. Two members of the research team attended each session, one acted as the facilitator/moderator (MK) and the other took notes (EC). An illustrator (JM) also attended the focus groups creating real-time illustrations of key ideas and concepts that were shared on screen with participants during the session. These illustrations have been provided as an additional file [see Additional file 1].

### Data analysis

Data were transcribed, pseudonymised, and analysed using the Framework Method [28]. Data collection and data analysis proceeded iteratively. The video recording of the first interview was watched and listened to, and the transcript read, and re-read line by line by two analysts (EC and MK) who independently noted their initial thoughts and impressions of the data. Each analyst coded the transcript by applying a phrase or ‘code’ to important passages relevant to the research aims in the left-hand margin of the transcript. More detailed reflexive notes and explanations were made in the right-hand margin. A third analyst (TP) watched and listened to the video recordings of the sessions and provided written comments about important concepts related to the research aims. An initial analytical framework based on the analysis of the first analyst (EC) was then devised. The framework was pre-loaded with codes that were derived deductively based on our previous PPIE. The parallel inductive approach permitted the researchers to add unanticipated codes to the framework that were inferred directly from the data. Eight codes were initially identified and 31 subcodes. A brief description of each code provided summary information to enhance the transparency of the coding process.

The initial analytic framework was then compared to the completed coding of the same transcript undertaken by the second analyst (MK), and the written notes of the third analyst (TP). Minor adjustments were made. This second iteration of coding was then agreed as the working analytic framework and applied to subsequent transcripts. New overarching categories and codes that supplemented the working analytic framework were noted and agreed by the first (EC) and second (MK) analysists. Conceptually related codes were revised and grouped together. Summary information was edited to improve the description of each code and further examples of data that could be incorporated under each code were included in the description. A revised framework was devised to incorporate the new codes and categories. The iterative process of coding, revising and grouping continued until no new codes or categories were generated. The final framework consisted of six codes clustered into 40 subcodes.

The data were summarised in a spreadsheet matrix using Microsoft Excel. Data from the manuscripts were charted into the matrix, using one column per focus group or interview and one row per code. Direct quotations with line and page number references for each code were added to the matrix. Illustrative and interesting quotations were highlighted. Illustrations (that were created during the sessions) that represented the codes and quotations were added to the matrix. Illustration selection was agreed by both analysts. Narrative summaries were composed, and exemplar quotations were identified for all categories. The final themes and subthemes were agreed by both analysts (EC and MK) by reviewing the data matrix framework and connecting conceptually related ideas and categories. A pragmatic approach was adopted in finalising the themes to inform construction of the resource pack. Convergent and divergent views were retained for exploration with PPIE and the co-production team. This likely symbolises a lack of saturation of data. This study did not aim to reach saturation, given the subject of A–T which is a rare condition. However, by our third focus group there were no new themes regarding content raised compared to focus group 1 or 2. This provided some assurance to proceed with the first version of the pack.

## Results

Eight parents (6 mothers and 2 fathers) and two children with A–T (boy, aged 8 years and girl, aged 13 years) participated with support from their parents. The duration of data collection was: interview (30 min), parent focus groups (50 min each group), children’s focus group (just under 30 min). Three themes and 21 subthemes were generated (Table 1). The findings offer an insight to the range of participants’ views and a sense of convergence and divergence.

**Table 1:** Themes and Subthemes

Themes	Subthemes
1. Accessing, managing, organising, and sharing information with others	1.1. Record keeping: working with professionals 1.2. Sharing information about A–T with relatives, friends, and lay people
2. Pack Content	2.1. About me 2.2. Who helps look after me? 2.3. How does A–T affect me? 2.4. What is A–T? 2.5. My exercise programme 2.6. My treatment 2.7. My appointments 2.8. My test results 2.9. My health 2.10. My research trials and interventions 2.11. My questions for my healthcare team 2.12. Letters from my healthcare team 2.13. My education, health, and care plan 2.14. Research and guidelines about A–T
3. Design features	3.1. Name of the pack 3.2. Format of the pack 3.3. Style 3.4. Organisation 3.5. Language options

Theme 1. Accessing, managing, organising, and sharing information with others

**1.1. Record keeping: working with professionals**

Parents with a child with A–T are sent countless documents, letters, reports, test results, appointments and assessments from healthcare, social care, and educational organisations. Parents described having little time to gather, organise or manage these records. All parents had tried to store documents in a file or similar and to keep it up to date. However, incomplete access to information, lack of time, as well as absence of a dedicated record-keeping resource made keeping track difficult:

*‘I would like to [...] just have a file and…just keep the history of it somewhere. I was trying to scan some of those letters into my computer, but I have to say I haven't been very good at it.’ [Mother, 4].*

Parents were aware of data protection principles but were nonetheless surprised that medical and educational records were not stored in a central database with access provided to families, healthcare professionals and teachers. Parents told us that healthcare professionals may have access to some documents but that it was rare for one person to have the full picture. School staff and educational specialists usually only had access to educational reports and assessments. Parents may have copies of some but not all documents and letters and were not always included in communication between the professionals working with their child:

*‘we have an immunologist that deals with [my child]. And she, she records all this documentation. But, for example, if we go to a neurologist, he is in a different hospital and he hasn’t … he hasn’t [got] this documentation. […] Everyone concentrates on his own area. […] The speech therapist [in clinic] had some contact with the speech therapist from school. […] I wasn’t involved.’ [Mother, 1].*

Parents’ narratives inferred that different professionals hold different pieces of the jigsaw that made up the full picture, but nobody held all the pieces at the same time:

*‘I can imagine it’s [the pack] useful from a clinical point of view, it’s very useful. And for us whenever you go to see whoever the specialist may be to be able to just hand something over, which is a useful summary of everything that happened until now.’ [Father, 2].*

Fragmentation of information contributed to inconsistent communication and incomplete record keeping between parents and the professionals involved in their child’s life. The idea of an information pack held by, designed, and managed by families to help manage some of the burden of storing and retrieving relevant records was warmly received. Parents thought the pack could support communication and conversations with professionals. However, some of them also acknowledged that it may add to their overall burden as the responsibility to update the pack would lie with the families. A simple system that did not create extra work for families was therefore considered a priority:

*‘… everything I’m saying [about what should be included in the pack] means it’s a lot of work for us to do. That’s why I was asking before if there was a centralised system that other people are using too because otherwise it just becomes like a huge job for us, you know, to keep it all updated.’ [Mother, 4].*

**1.2. Sharing information about A–T with relatives, friends, and lay people**

Parents also wanted to share information about A–T with friends, families, and lay people. However, they found it difficult to judge how to best share information without overwhelming for the recipient.

*‘I feel a bit uncomfortable then for the person who I’m talking to, because if you give the full explanation of what it [A–T] is and what it means and you know, it’s, it’s quite heavy to drop that on somebody. Whereas if you had maybe a less confronting way of sharing that information, then then yeah it just makes it … It skips those types of conversations… Finding that right balance of what information people should get at what point and what they’re ready to receive is key.’ [Father, 2].*

Being able to refer others to a trusted source of further information about A–T, such as the A–T Society, was thought to be an effective way of dealing with the information needs of friends, families, and lay people, and reduced the burden of having to repeatedly explain, share and manage information with different groups of people. Parents suggested that packs would need to be individual and populated by families according to what would work best for them and their child. Having a pack with a *“good lay summary” [Father, 1]* that could be shared with others, “*with a lot of information about A–T’ [Child, 1]* and which included signposting to reliable and accessible sources of information was thought to be potentially useful:

*‘… also [explain] what A–T is […] If nothing else, it would be much more time efficient than explaining to people who you meet what A–T is and what it means and what it means for [our child].’ [Father, 2].*

Whilst none of the children wanted to share the pack with their friends, they thought that sharing the pack with others, including adults involved in their care, as well as other children and young people with A–T would have some practical value. One child said the pack would be useful for professionals:

*‘Maybe the people that have A–T. And the parents that are having to keep children safe with A–T and help them maybe, with some advice. Maybe doctors that help children with A–T […] everyone.’ [Child, 2].*

Whereas the other child was keen for more people to know about A–T:

*‘Everyone who wants to learn about A–T. ‘[It’s for] everyone who would like to know more about my condition. […] I’m not worried if the information is shared with other people.’ [Child, 1].*

Theme 2. Pack content

Parents and children expressed views about what information should be contained in the pack. Specific sections are noted in Table 1. Children strongly advocated a person-first approach as they wanted the first pages of the pack to include an ‘About Me’ section with personal information such as their name, age, family, friends, pets, clubs, hobbies, and their likes and dislikes:

*‘My name, my age […], pictures of me […] … pictures with my friends […] My favourite things to do in my spare time. […] … people should know that everybody is equal.’ [Child, 1].*

The children were keen to be seen first and foremost as typical children for their age, living a happy and full life, rather than a child with a health condition:

*‘I hope that people with A–T have a happy life. […] ‘I’m very active. I do a lot of clubs.’ ‘… some writing about me. […] ‘You might need some help with your running and jumping […] things that might be trickier for everyone that has A–T.’ [Child, 2].*

The children also thought it would be useful to have a section about the people who helped look after them:

*‘About the doctors that you see. […] I see a bunch of nurses so maybe we could put them in there?’ [Child, 2].*

Consistent with the record-keeping purpose of the pack, and in addition to including information about A–T, parents and children suggested that a healthcare section would be useful with subdivisions on current treatments and interventions, test results, appointments, letters, other health conditions and illnesses, and a section on allergies and specific dietary preferences such as information about “*which doctors you might want to go to…what medicine you might need” [Child, 2].* Parents supported the importance of record keeping:

*‘… definitely I would say medication, what they are taking with dates, reason why… when they are ill, how they’re ill. Is it a temperature, cough, or chest infection or whatever diarrhoea, and other things that can happen.’ [Mother, 4].*

Exercise programmes prescribed by physiotherapists were also seen by parents and children as being an important component of the pack, to show:

*‘how important exercise is, … some pictures of exercises that people might want to do.’ [Child, 1].*

Another factor related to tracking effectiveness:

*‘[the child] has exercises with the teacher [at] school and with therapists at the [clinic]. And in this pack maybe, we will be able to trace which of them is more effective and which is not because not all exercises change anything.’ [Mother, 1].*

Parents also described other content that could add to the record-keeping and information storage purposes of the pack. These suggestions included adding in sections on research and involvement in medical trials, for example:

*‘I’d love to see what sort of therapies are out there for things like breathing and lung capacity and all that kind of stuff. It’s not something we always find the time to do…I’d welcome that” [Father, 1].*

Having access in one place to relevant guidelines on A–T was considered helpful:

*‘ …[having] them in one place.. you know where to find everything, if you need to look into something or consult with a doctor like you have it in a meeting and you have that with you.’ [Mother, 2].*

Another suggested section related to questions for members of their child’s healthcare team and storage of the Education, Health, and Care plan (EHCP):

*‘… the EHC plan would probably be good to have in there.’ ‘We’ve got appointments with schools to talk about EHCP and any changes happening. […] … there might be a slight change or bigger change happening at school that's medically related, so it's good to keep track of that.' [Mother, 4].*

Theme 3. Design features

Key design features are noted in Table 1. The children were keen to personalise the pack by naming it, for example Child 1 suggested it should be called the ***“[my name]'s A–T Pack?***' and Child 2 agreed “*Yeah, I love that*”. Children preferred the idea of the pack being “*not too big*” *[Child, 1]*, “*a thing that you can hold in your hands, like a physical object” [Child, 2]*, like a “*magazine” [Child, 1].* They also proposed it should be colourful and engaging:

*'… with a lot of pictures and a lot of different colours.’ [Child, 1].*

The addition of customised features and stickers that expressed their interests was thought to be important:

*'Maybe some stickers that have a smiley face on them and som’e, some, food stickers. Because I like food. Who doesn’t? And I like dogs yeah, only dogs that don’t bark.’ [Child, 2].*

In contrast to the children’s preference for a hand-held paper copy, parents expressed a preference for an electronic version, possibly in the form of an app that would in the future be available in different languages. Parents also acknowledged the importance of making the pack reader friendly for lay people so as to *“really gently ease people in…[so that] within a few minutes [a layperson] can understand what A–T is” [Father, 1]*. Parents thought this could be achieved by breaking up the text:

*‘…you need to make it look interesting, is not like big lessons and big essays and nobody reads that. […] family and friends, they just look at highlights and also, they don't go into the details to read about it.’ ‘… just the points that they can quickly read it and understand quickly what is. So that will be much more kind of easy to pick up.’ [Mother, 5].*

## Discussion

This qualitative study has co-produced the first family-owned information pack for CYP with A–T. The pack was drafted and illustrated with the assistance of a graphic designer who attended the foc’us group sessions. Two versions of the pack were created: a paper and a digital format, providing flexibility of choice.

Study participants highlighted the potential for an A–T specific family-owned healthcare pack to contribute to more effective record keeping, symptoms management, and information sharing with relevant healthcare and other professionals involved in children’s care. Parents noted they struggled to manage countless documents and information related to their child’s health and were concerned by the lack of an accessible centralised system that contained all the information about their child’s health and care. Although patient healthcare records (PHRs) are reported to promote patient self-management and greater involvement in their own care [21], existing examples are limited in their scope due to their ownership and use mainly being controlled by the healthcare providers [19, 24].

The discussion now considers the perspectives of children with A–T and parents of CYP with A–T about the utility, acceptability, design, and content of the family-owned pack.

### Utility

The parents and children found the proposed pack valuable as an information sharing and record keeping tool. They considered it useful as a means of sharing information about A–T and its impact with friends, family, and lay people. The children felt excited by the idea of owning a pack that they could hold and share as it would minimise the need for conversations with other people, as they reported in the focus group session that this was a topic that they did not like talking about with others. The pack would also enable others to know more about’ their interests, likes and dislikes rather than focusing solely on the medical condition, which is one aspect of their life, and should not define their entire identity. It is essential to understand children as unique individuals, and this was echoed by the children who participated in this study. This has been seen in other work too that shows that CYP and their parents don’t want to be defined by a diagnosis [29–31].

Different from the children’s need to tailor the pack to communicate who they are as a person, parents reported needing a data management/organising system to help them navigate and use information more effectively. Parents acknowledged the need for an organised and coordinated approach to managing documents containing important information related to their child’s health and development, a need that has received considerable attention among investigators [32, 33]. Until a centralised digital platform is implemented that allows CYP and their parents to continuously (and not just upon request) view, edit, and monitor health and educational records and assessments, the A–T pack has the potential to provide a viable option to cope with the struggles of record keeping.

The pack has the potential to: a) track symptoms and treatment during and following an illness to manage it better in the event of recurrence, b) document therapeutic interventions that a child may be involved in to assess their effectiveness, and c) record a diet plan to maintain diet quality and nutritional intake. This will enable people with A–T and their families to manage aspects of their physical and mental health affected by recurring infections, chronic fatigue, and poor nutritional status.

Some of the parents were concerned by the lack of coordination between the different professionals involved in their child’s care, an issue often highlighted by parents/caregivers of children with long-term health conditions [34–37] exacerbating the time demands on families [38] and adding to their overall burden [36]. Collaborative relationships between families, healthcare professionals, staff at school, educational specialists, and any other professionals that may be involved in caring for a child, was identified as key and efforts should be made to cultivate this. The A–T pack that has been co-produced in this study, is expected to provide better coordination of assessments and appointments between different professionals involved in caring for a child with A–T.

### Acceptability

The idea of owning a pack as a record keeping and information sharing resource was welcomed by parents and children who participated in this study. This was viewed as a step towards improving quality of life, which is considered important alongside the efforts of finding a cure for the condition [39].

Despite the overall acceptance of the pack and its utility in general, some parents expressed concerns about maintaining the pack. They had a positive view of the pack if it did not add to their overall burden of managing life with a child with a long-term health condition. Due to the dynamic nature of the content of the pack, it would need to be updated regularly and the responsibility of doing that would lie with the parents. The parents expressed a strong need for a digital centralised system where all professionals working with their child(ren) can add notes and update reports, so that the burden of maintaining an up-to-date record of the child’s health and education doesn’t lie solely with the families. Ultimately a centralised system with secure access could provide a useful way forward allowing professionals to log in an update as care is delivered. However, until such system is designed and implemented, this pack could provide parents with a simple and quick way of storing and retrieving important information.

### Design

Children and parents had different views about the design of the pack. While children pictured the pack as a magazine style booklet to hold and carry around, parents wanted the pack to be electronic or an app that they can edit and easily maintain with simultaneous, remote access. This debate between paper and electronic versions of healthcare records has long existed, with many authors exploring the comparisons between the quality of two in terms of content, process and structure [40], efficiency in record keeping and physician satisfaction in the emergency department [41], and the quality of documentation in mental health centres [42]. Advances in technological innovation in the last decade, have resulted in a variety of ways that individuals, groups, and organisations can store and retrieve information [43, 44]. In healthcare delivery, the electronic medical records have become an integral part, especially in developed countries [41, 45, 46].

As a potential solution to accommodate the different preferences expressed by the children and the parents, the pack has two sections. Sect. “Background” focuses on sharing general information about A–T and the child and may be held as a paper version. This is the section that children are more likely to use and carry around with them and has been designed to look more visually appealing with the use of colours, pictures, and space for adding stickers, in line with the children’s preferences. Sect. “Methods” caters more to the needs of the parents, with scope for organising, managing, saving and amending information electronically. This section will require regular updating and includes sub-sections on their child’s symptoms, illnesses, therapies, appointments, and EHCP.

### Content

Parents and children had clear ideas about what should be included in the pack, based on what would help them in managing care. There were no conflicting opinions between the parents, or between the parents and the children; and they all built upon each other’s ideas. The pack has the potential to serve a dual purpose, storing and retrieving information, and helping to communicate and work with other professionals involved in providing care to their child.

However, although it is important to identify information that may be helpful to children and their parents, there is also a need to find the right balance. Information needs to be presented in an accessible format using simple language for young readers or lay people [47]. Additionally, since A–T is a progressive condition, a major consideration is that the children who own and use this pack will be of different ages and experiencing different manifestations of A–T (such as dependency on wheelchair, pubertal delay, etc.). The pack needs to address this range of information whilst not overwhelming lay readers as well as the children and parents it is designed to support.

It is acknowledged that some sections relating to tracking and storing information about care and management of A–T, may not be helpful or required by all the parents, therefore the digital format of Sect. “Methods” of the pack will allow families to adjust the content of the pack based on their own requirements.

### Strengths and limitations

The main strength of this study is the person-centered approach [48], which focused on eliciting the views of CYP and their families in co-production of an A–T specific family-owned healthcare pack. Despite the consensus that CYP (and their parents) have the right and should be encouraged to be fully involved in decisions about their care [19, 23], to date, no studies have actively consulted them in the development of PHRs, the benefits of which are discussed, and its adoption is recommended in several studies [19, 21, 49]. This study involved children with A–T and parents of CYP with A–T in the development of a healthcare pack with the view to explore its utility and acceptability, while giving families control over the content of the pack.

Another strength of this study is related to its methodological quality and the use of an illustrator creating real-time illustrations and ‘minutes’ during the pilot interview and focus group sessions. This helped the parents and children to visualise the ideas that came up during the discussions and helped to clarify whether their views were communicated and interpreted effectively. The real-time illustrations also made the discussions more engaging and fun for the children who enjoyed looking at the drawings, as seen in other studies [50, 51]. The illustrations produced during the sessions were member checked with all the participants after each session. This was done by sending them the illustrations and requesting their feedback on whether they accurately communicated and interpreted their ideas and views and resonated with their experiences. The final design of the pack was also shared with children and parents who participated in this study to check its relevance and accuracy before its wider dissemination.

Despite its strengths, the study has limitations, primarily related to the small sample size and in turn uncertainty regarding representation of the broad spectrum of experiences of CYP with A–T. This is a common challenge in research with people living with rare conditions [52, 53]. Although all eligible families in the UK were approached in an effort to recruit a representative sample, only two CYP participated directly in the study and as such their detailed demographics cannot be disclosed in order to maintain confidentiality. This limitation necessitates cautious interpretation and application of the results to wider contexts.

While the factors contributing to the low participation rates could not be fully explored within this study, common features associated with A–T such as fatigue [54] and speech-related challenges [55], may have been significant barriers despite the accessible online design. It was clear to the research team that the parents often contributed a proxy voice as they commented that they had discussed and explored their children’s perspective before the focus group. It is the research team’s experience that parents similarly often provide this proxy voice in clinical settings for their children with communication difficulties.

### Pack dissemination

Feedback on early pack design and the final paper version was sought from the families who participated (n = 11) in the wider ATeam Collaborative programme of work (detailed at: osf.io/edzn3/). New copies or additional pages can be requested from the team via an automated weblink (tinyurl.com/bh2erubf). Whilst version 1 of this pack was coproduced by participants who consented to partake in research, a Microsoft form, embedded in the pack design, provides a mechanism for ongoing evaluation; allowing families to share their feedback on the pack ahead of future refinement. Due to limited funding, the availability of free paper copies is restricted, but families can request unlimited digital copies or chose to self-fund their own printed copy.

### Implications for further research

Future research will engage the wider international A–T community and communities of CYP with other rare a’taxia types. This original pack design could then be adapted to meet the needs of these populations. This future research will rigorously assess the usability and impact of the pack internationally and guide future refinement for maximal impact.

Whilst beyond the scope of the curent funded research, future studies could also explore the development of an app. This was identified as an important feature by parents and could allow for real-time updates and remote sharing of information among families and professionals. Such an app could enhance connectivity and support for families and start to address the bigger problem of fragmented information for healthcare providers working across multiple healthcare systems [56]. Access to all complex health information in one place would foster transparency and aid collaboration between healthcare providers and families. This could play a critical role in breaking down barriers and lead to an improved care experience for CYP and their families [57].

## Conclusion

The development of this pack marks a significant step towards supporting families in organising and managing complex health information and communication. Continued refinement and evaluation will be crucial in ensuring that it meets the evolving needs of CYP with A–T and their families and other care providers.

Healthcare systems nationally and internationally should work towards establishing patient centered electronic health records to streamline communication and coordination among the family and professionals involved in the child’s care. Until this is fully implemented, resources such as this pack can help to break down barriers and improve communication to enhance family care experiences in complex and rare conditions.

## Supplementary Information

Additional file1

## Data Availability

The illustrations produced during the pilot interview and focus groups are included in this published article as additional file 1 in a pdf format. Other datasets used and/or analysed during the current study are available from the corresponding author on reasonable request.

## Abbreviations

A–T: Ataxia telangiectasia
PHR: Patient-held health record
PPIE: Patient and public involvement and engagement
NHS: National health service
CYP: Children and young people
EHCP: Education, health, and care plan
FG: Focus group
