# Supplementary material for: My A–T pack: a qualitative study of the utility, acceptability, design, and content of a family-designed and owned information pack relevant to the lives of children and young people living with ataxia telangiectasia
Source: Orphanet J Rare Dis. 2025 Aug 4;20:397. doi: 10.1186/s13023-025-03919-6 (PMC12323091; doi:10.1186/s13023-025-03919-6)
Supplement: Supplementary file 1 — Additional file1 [file 13023_2025_3919_MOESM1_ESM.pdf]

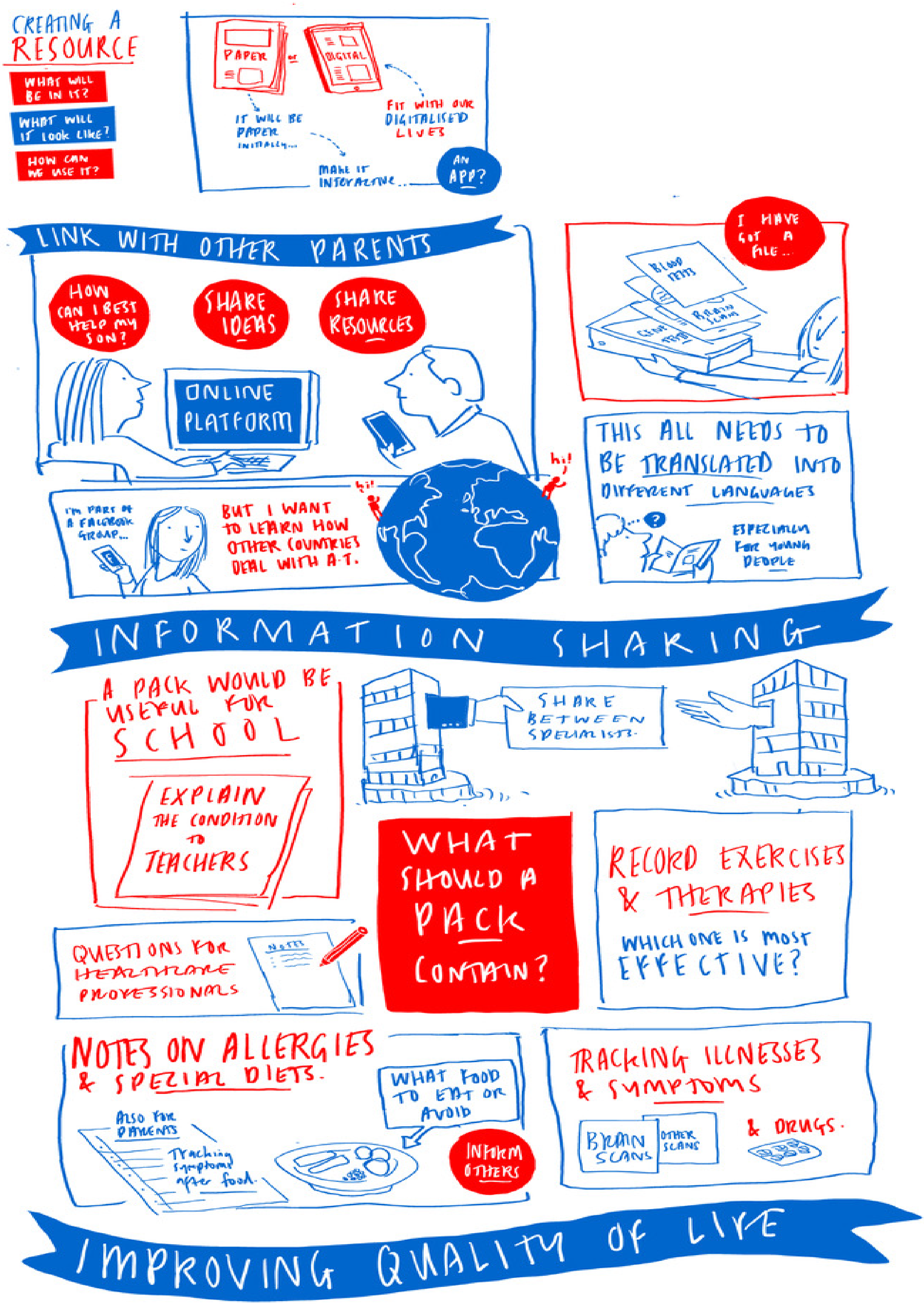
CREATING A RESOURCE
WHAT WILL BE IN IT?
WHAT WILL IT LOOK LIKE?
HOW CAN WE USE IT?
PAPER
OR
DIGITAL
IT WILL BE PAPER INITIALLY...
FIT WITH OUR DIGITALISED LIVES
MAKE IT INTERACTIVE...
AN APP?
LINK WITH OTHER PARENTS
HOW CAN I BEST HELP MY SON?
SHARE IDEAS
SHARE RESOURCES
ONLINE PLATFORM
I HAVE GOT A FILE...
BLOOD TESTS
BRAIN SCAN
THIS ALL NEEDS TO BE TRANSLATED INTO DIFFERENT LANGUAGES
ESPECIALLY FOR YOUNG PEOPLE
hi!
hi!
I'M PART OF A FACEBOOK GROUP...
BUT I WANT TO LEARN HOW OTHER COUNTRIES DEAL WITH A-T.
INFORMATION SHARING
A PACK WOULD BE USEFUL FOR SCHOOL
EXPLAIN THE CONDITION TO TEACHERS
SHARE BETWEEN SPECIALISTS.
WHAT SHOULD A PACK CONTAIN?
RECORD EXERCISES & THERAPIES
WHICH ONE IS MOST EFFECTIVE?
QUESTIONS FOR HEALTHCARE PROFESSIONALS
NOTES
NOTES ON ALLERGIES & SPECIAL DIETS.
ALSO FOR PARENTS
Tracking symptoms after food.
WHAT FOOD TO EAT OR AVOID
INFORM OTHERS
TRACKING ILLNESSES & SYMPTOMS
BRAIN SCANS
OTHER SCANS
& DRUGS.
IMPROVING QUALITY OF LIFE

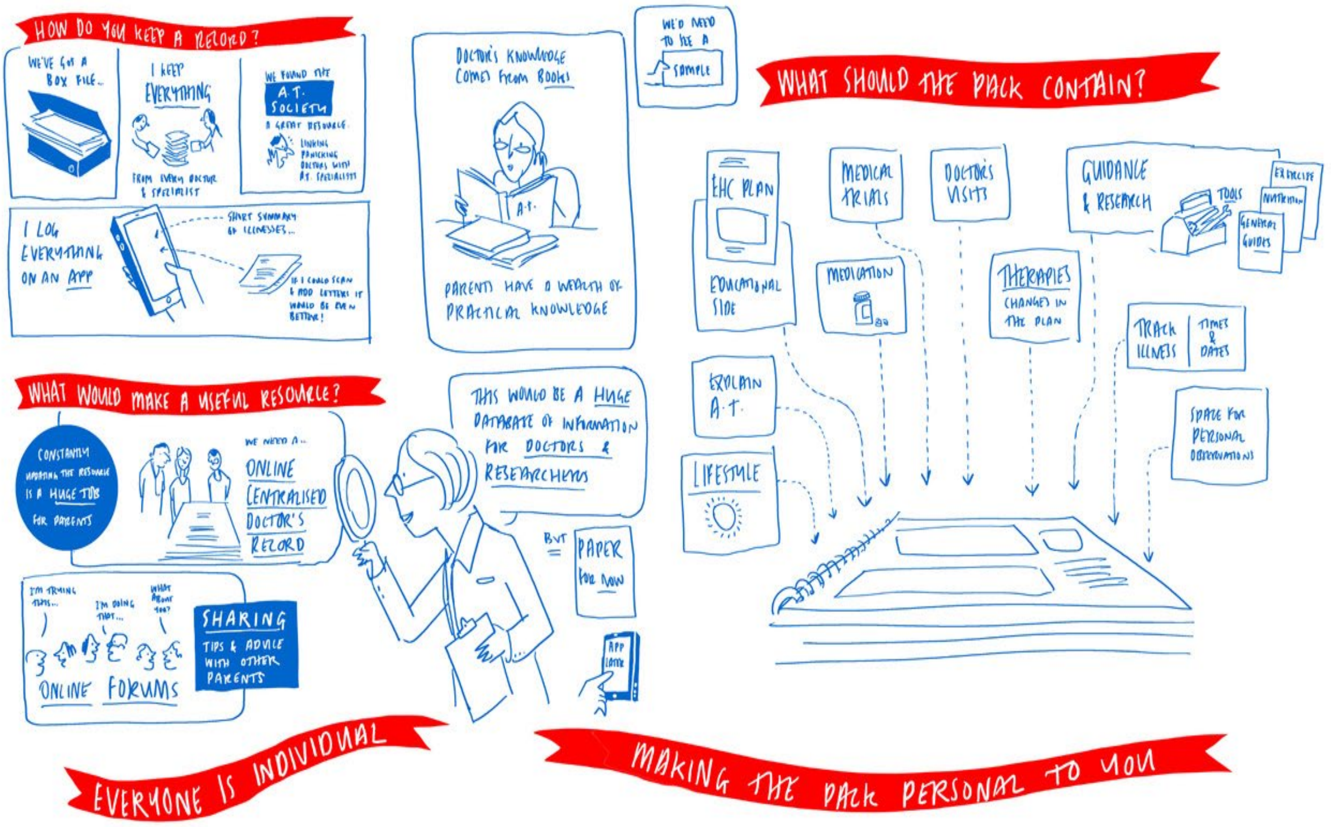

HOW DO YOU KEEP A RECORD?
WE'VE GOT A BOX FILE...
I KEEP EVERYTHING
FROM EVERY DOCTOR & SPECIALIST
WE FOUND THE A.T. SOCIETY A GREAT RESOURCE.
LINKING PANICKING DOCTORS WITH A.T. SPECIALISTS
I LOG EVERYTHING ON AN APP
SHORT SUMMARY OF ILLNESSES...
IF I COULD SCAN & ADD LETTERS IT WOULD BE EVEN BETTER!
DOCTOR'S KNOWLEDGE COMES FROM BOOKS
A.T.
PARENTS HAVE A WEALTH OF PRACTICAL KNOWLEDGE
WE'D NEED TO SEE A SAMPLE
WHAT SHOULD THE PACK CONTAIN?
EHC PLAN
EDUCATIONAL SIDE
MEDICAL TRIALS
DOCTOR'S VISITS
GUIDANCE & RESEARCH
TOOLS
EXERCISE
NUTRITION
GENERAL GUIDES
MEDICATION
THERAPIES
CHANGES IN THE PLAN
TRACK ILLNESS
TIMES & DATES
EXPLAIN A.T.
SPACE FOR PERSONAL OBSERVATIONS
LIFESTYLE
WHAT WOULD MAKE A USEFUL RESOURCE?
CONSTANTLY UPDATING THE RESOURCE IS A HUGE JOB FOR PARENTS
WE NEED A...
ONLINE CENTRALISED DOCTOR'S RECORD
THIS WOULD BE A HUGE DATABASE OF INFORMATION FOR DOCTORS & RESEARCHERS
BUT = PAPER FOR NOW
APP LATER
I'M TRYING THIS...
I'M DOING THAT...
WHAT ABOUT YOU?
ONLINE FORUMS
SHARING TIPS & ADVICE WITH OTHER PARENTS
EVERYONE IS INDIVIDUAL
MAKING THE PACK PERSONAL TO YOU

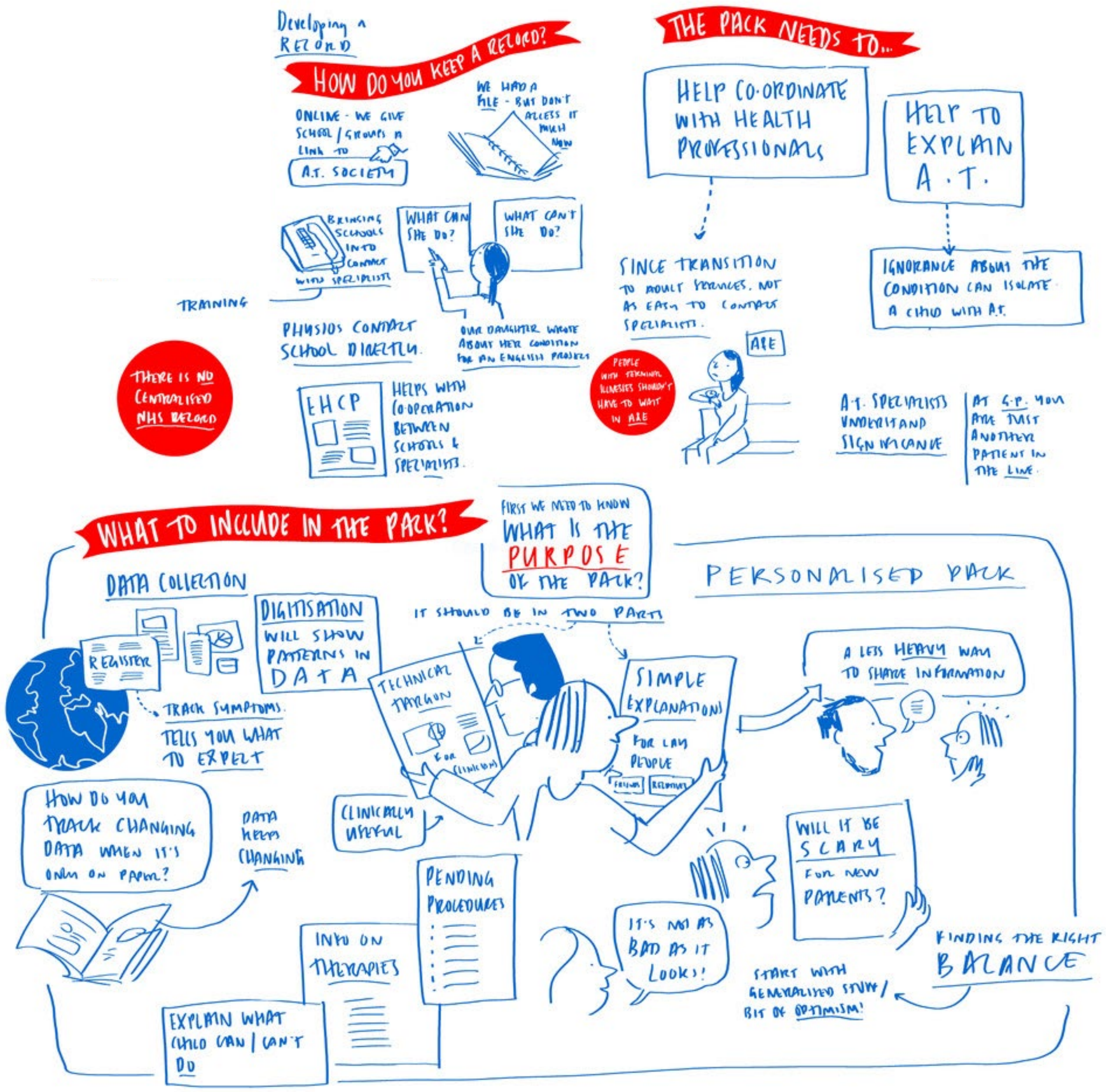

Developing a RECORD
HOW DO YOU KEEP A RECORD?
ONLINE - WE GIVE SCHOOL / GROUPS A LINK TO
A.T. SOCIETY
WE HAD A FILE - BUT DON'T ACCESS IT MUCH NOW
BRINGING SCHOOLS INTO CONTACT WITH SPECIALISTS
WHAT CAN SHE DO?
WHAT CAN'T SHE DO?
TRAINING
PHYSIOS CONTACT SCHOOL DIRECTLY.
OUR DAUGHTER WROTE ABOUT HER CONDITION FOR AN ENGLISH PROJECT
THERE IS NO CENTRALISED NHS RECORD
EHCP
HELPS WITH CO-OPERATION BETWEEN SCHOOLS & SPECIALISTS.
THE PACK NEEDS TO...
HELP CO-ORDINATE WITH HEALTH PROFESSIONALS
HELP TO EXPLAIN A.T.
SINCE TRANSITION TO ADULT SERVICES, NOT AS EASY TO CONTACT SPECIALISTS.
IGNORANCE ABOUT THE CONDITION CAN ISOLATE A CHILD WITH A.T.
PEOPLE WITH TERMINAL ILLNESSES SHOULDN'T HAVE TO WAIT IN A&E
A&E
A.T. SPECIALISTS UNDERSTAND SIGNIFICANCE
AT G.P. YOU ARE JUST ANOTHER PATIENT IN THE LINE.
WHAT TO INCLUDE IN THE PACK?
FIRST WE NEED TO KNOW WHAT IS THE PURPOSE OF THE PACK?
PERSONALISED PACK
DATA COLLECTION
REGISTER
DIGITISATION WILL SHOW PATTERNS IN DATA
TRACK SYMPTOMS. TELLS YOU WHAT TO EXPECT
IT SHOULD BE IN TWO PARTS
TECHNICAL JARGON
FOR CLINICIANS
SIMPLE EXPLANATIONS FOR LAY PEOPLE
FRIENDS RELATIVES
A LESS HEAVY WAY TO SHARE INFORMATION
HOW DO YOU TRACK CHANGING DATA WHEN IT'S ONLY ON PAPER?
DATA KEEPS CHANGING
CLINICALLY USEFUL
PENDING PROCEDURES
INFO ON THERAPIES
EXPLAIN WHAT CHILD CAN / CAN'T DO
WILL IT BE SCARY FOR NEW PATIENTS?
IT'S NOT AS BAD AS IT LOOKS!
START WITH GENERALISED STUFF / BIT OF OPTIMISM!
FINDING THE RIGHT BALANCE

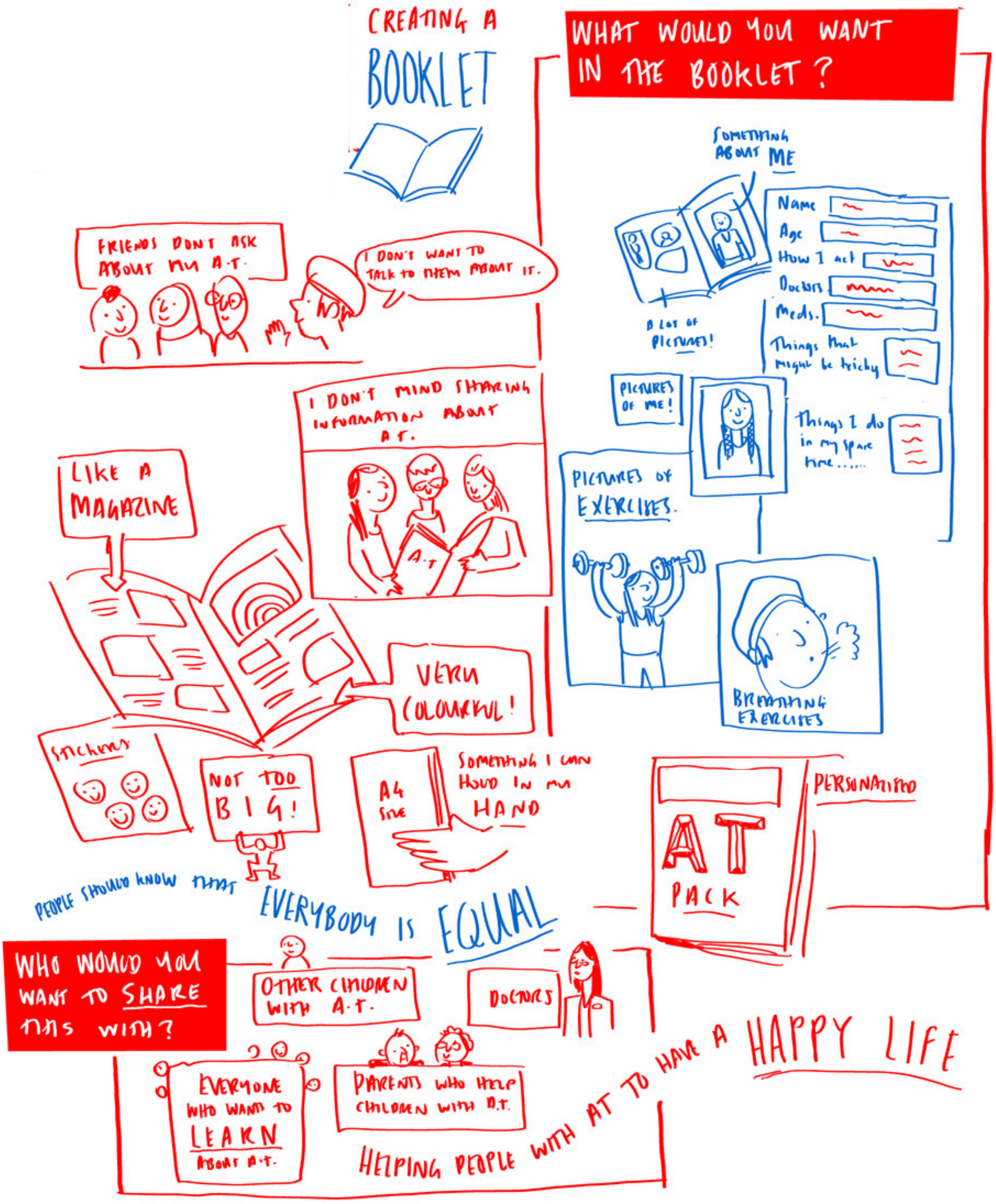
CREATING A
BOOKLET
WHAT WOULD YOU WANT IN THE BOOKLET?
SOMETHING ABOUT ME
Name
Age
How I act
Doctors
Meds.
Things that might be tricky
A LOT OF PICTURES!
PICTURES OF ME!
Things I do in my spare time.....
PICTURES OF EXERCISES.
BREATHING EXERCISES
FRIENDS DON'T ASK ABOUT MY A.T.
I DON'T WANT TO TALK TO THEM ABOUT IT.
I DON'T MIND SHARING INFORMATION ABOUT A.T.
A.T
LIKE A MAGAZINE
VERY COLOURFUL!
STICKERS
NOT TOO BIG!
A4 SIZE
SOMETHING I CAN HOLD IN MY HAND
PERSONALISED
AT PACK
PEOPLE SHOULD KNOW THAT EVERYBODY IS EQUAL
WHO WOULD YOU WANT TO SHARE THIS WITH?
OTHER CHILDREN WITH A.T.
DOCTORS
EVERYONE WHO WANTS TO LEARN ABOUT A.T.
PARENTS WHO HELP CHILDREN WITH A.T.
HELPING PEOPLE WITH AT TO HAVE A HAPPY LIFE
